# Limb lengthening history, evolution, complications and current concepts

**DOI:** 10.1186/s10195-019-0541-3

**Published:** 2020-03-05

**Authors:** Gamal A. Hosny

**Affiliations:** Benha University Hospitals, 11 Al Israa Al-Mohandeseen Street, Cairo, Egypt

**Keywords:** Limb lengthening, Indications, Achondroplasia, Cosmetic lengthening, Upper extremity lengthening

## Abstract

Limb lengthening continues to be a real challenge to both the patient and the orthopaedic surgeon. Although it is not a difficult operative problem, there is a long and exhausting postoperative commitment which can jeopardize early good results. I aim to review the history, evolution, biology, complications and current concepts of limb lengthening. Ilizarov’s innovative procedure using distraction histeogenesis is the mainstay of all newly developing methods of treatment. The method of fixation is evolving rapidly from unilateral external fixator to ring fixator, computer assisted and finally lengthening intramedullary nails. The newly manufactured nails avoid many of the drawbacks of external fixation but they have their own complications. In general, the indications for limb lengthening are controversial. The indications have been extended from lower limb length inequality to upper extremity lengthening, including humeral, forearm and phalangeal lengthening. A wide range in frequency of complications is recorded in the English literature, which may reach up to 100% of cases treated. With developing experience, cosmetic lengthening has become possible using external or internal lengthening devices with an acceptable rate of problems.

**Level of evidence:** V.

## Introduction and history

Alessandro Codivilla of Bologna was the first to apply skeletal traction for bone lengthening. He used acute forced lengthening for short distances under narcotics. He described another technique for larger distances by continuous extension, using distraction with a calcanean pin and oblique osteotomy, followed by traction of 25–30 kg. More lengthening can then be achieved by applying more traction in stages [1]. One-stage lengthening was developed by Fassett using an osteotomy, inserting a bone graft and fixating with a plate. However, this procedure was followed by many serious complications [2].

In 1932, Abbot presented his experience with lower limb lengthening of 73 patients (45 tibial lengthenings) at the Shriners’ Hospital for Crippled Children in St. Louis. The basic principles stated in this paper were traction and counter traction through the bone, slow continuous traction to overcome the resistance of the soft tissues and accurate contact and alignment of bone ends. He described in detail the basic principles of tibial lengthening, including the application of two pins above and below the osteotomy, connected to a special apparatus. The drill pins were made of stainless steel, not ordinary steel, as it is less irritating to the soft tissues. The operative steps were: lengthening of the Achilles tendon, osteotomy of the fibula, insertion of the pins, application of the apparatus, osteotomy of the tibia and closure of the wound with drainage. Tibial osteotomy had to be performed with minimal soft tissue dissection to keep the blood supply to the bone and guard against infection. The surgeon had to wait for 1 week until the swelling had gone down before distraction. This was the first description of the waiting period before the Ilizarov era. The average distraction rate was 1.6 mm per day and the period of traction was 4 to 5 weeks. The apparatus was kept in place for 8 to 10 weeks followed by removal and application of a plaster cast. Follow-up X-rays were taken every 2 to 3 weeks to check the bone formation. The age of patients ranged from 8 to 19 years. The magnitude of tibial lengthening ranged from 3.81 to 8.89 cm. They reported excellent results with tibial lengthening but less favourable results with the femur and a higher rate of complications [3]. Then, Dickson and Diveley reported on an apparatus that used Kirschner wires rather than larger diameter pins to minimize soft tissue damage [4]. The method developed by Wagner gained popularity in Europe and the US; the method consisted of 3 operations. The first operation was the application of unilateral external fixation and a diaphyseal osteotomy. There was no waiting period, so acute operative lengthening for 5 mm was performed, followed by daily distraction of about 1.5 mm. The second operation was plating and bone graft. The third operation was plate removal and casting. However, a high rate of complications was recorded [5, 6].

Most of our contemporary knowledge of bone lengthening comes from the Ilizarov method. Ilizarov started his work in 1951 by treating a patient with a bone defect using a circular frame and transfixation tensioned wires. Then he discovered the biological law of tension stress or distraction histeogenesis and applied this principle to treat a wide variety of conditions such as nonunion, osteomyelitis, dwarfism, congenital deformities, some bone tumours, bone defects, fractures and bone shortening [7]. Recently, hexapodal computer assisted circular frames such as the Taylor Spatial Frame have gained in popularity. The next step in development was the application of self distraction motorized nails (a magnetically driven, titanium intramedullary nail) to avoid the complications of external fixation and gain rapid rehabilitation. However, Ilizarov’s principles still are the cornerstones of all bone lengthening procedures.

## Biology of limb lengthening

**Fig. 1:**
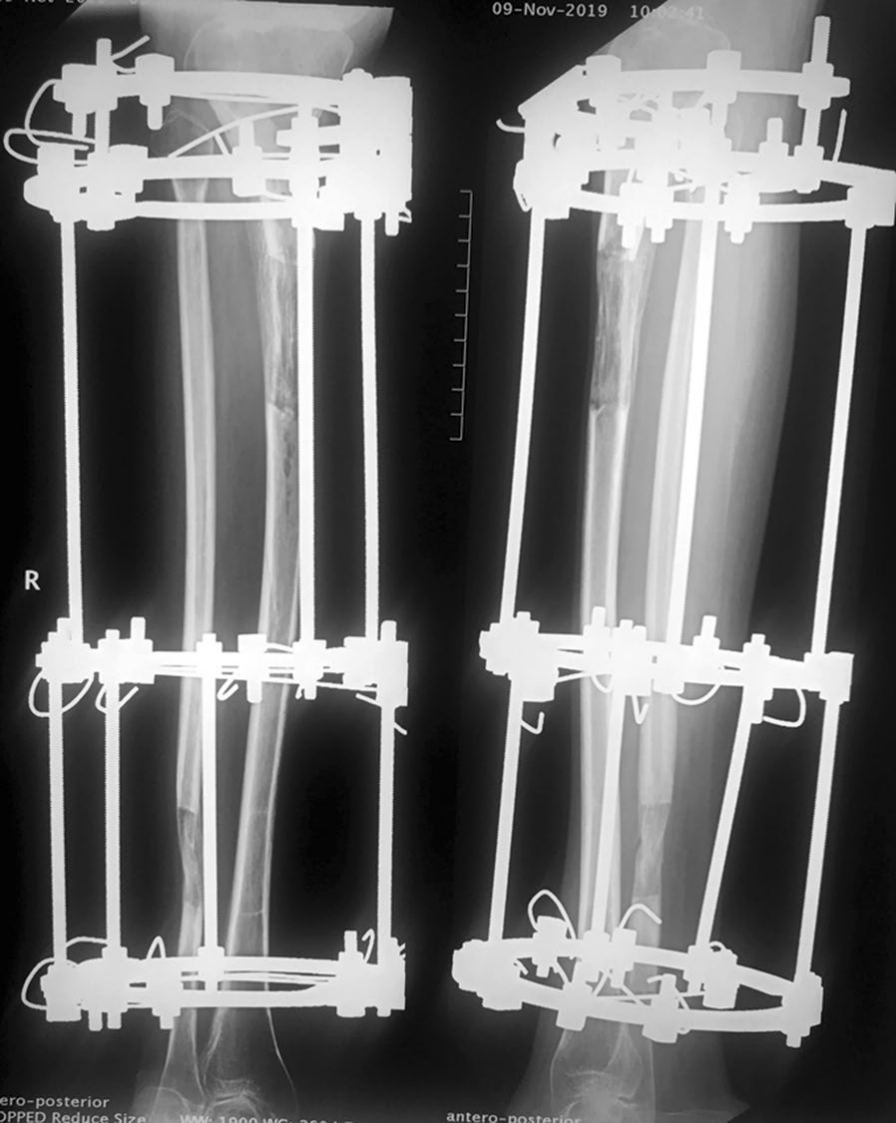
Tibial lengthening case showing that the regeneration develops along the axis of the applied traction

The current basic principles of bone lengthening are derived from the general biological law of tension stress. Gradual traction on living tissues creates stresses that can stimulate and maintain the regeneration of active growth of certain tissues. With adequate blood supply, steady gradual traction of the tissues activates proliferative and biosynthetic functions. The regeneration develops along the axis of the applied traction (Fig. 1). Experimental studies revealed the importance of soft tissue preservation during corticotomy and fixator stability, and the osteogenic power in the regeneration area depends upon the degree of bone marrow damage, periosteum and nutrient vessels. With distraction, new blood vessels develop in the transverse or longitudinal direction according to the tension vector. Under the tension-stress effect neovascularization occurs not only in bone but also in the soft tissue [8–10].

**Fig. 2:**
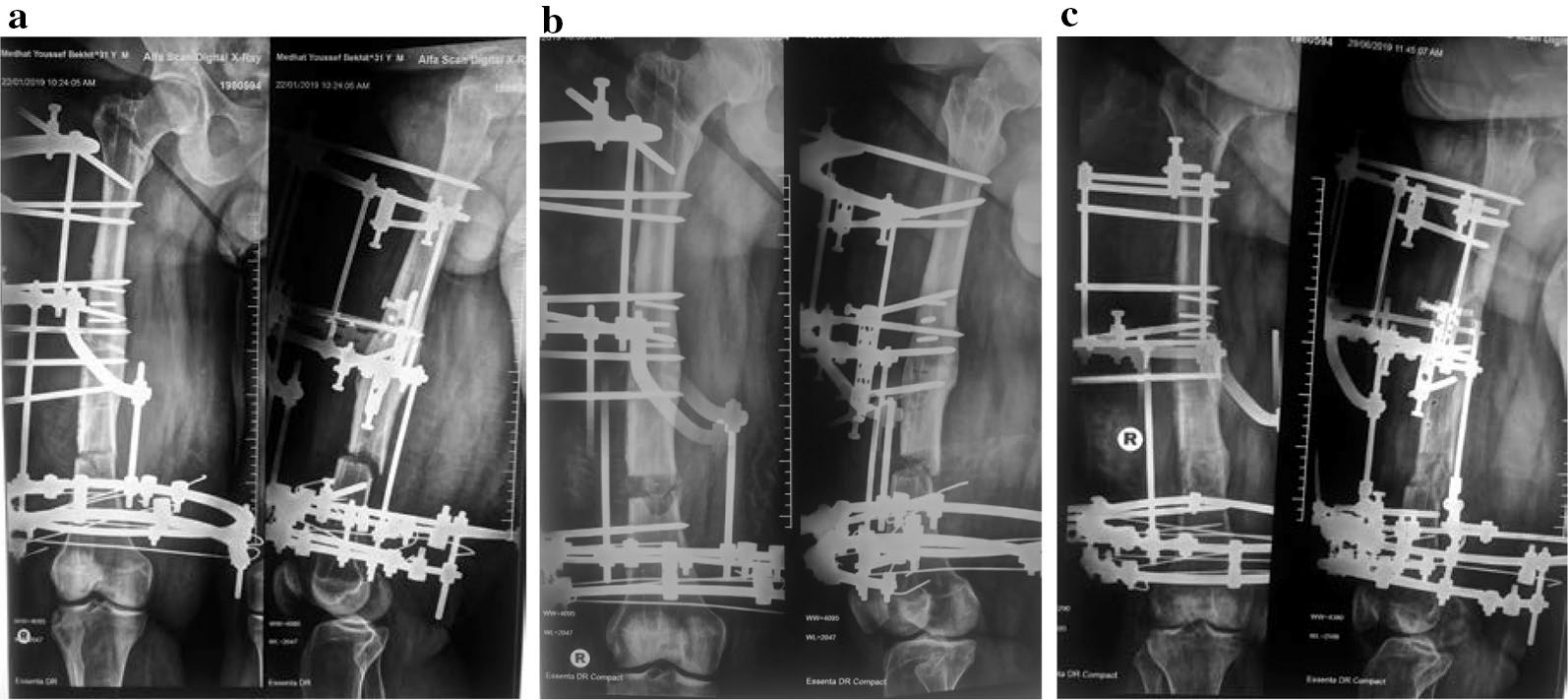
**a** Femoral lengthening case during the latency phase after corticotomy. **b** Distraction phase. **c** Consolidation phase

The biology of bone lengthening includes 3 stages: the latency phase, the distraction phase and the consolidation phase (Fig. 2). The process starts with a corticotomy, which is similar to a closed low-energy fissure fracture, and secure fixation of the two ends. Distraction can be of the callus or physis according to the site of application of the tension-stress effect [11].

The action of all types of external fixators, whether unilateral or circular, and internal medullary lengtheners, are based upon the law of tension stress [12, 13].

Stimulation of the regeneration area by systemic or local measures has been the mainstay of experimental and clinical research to enhance callus formation and decrease the time the fixator needs to remain in place. Systemic administration of bisphosphonates, high doses of alendronates, calcitonin and nerve growth factor have been administered in experimental trials with variable degrees of success. Local augmentation using a wide range of cells and growth factors such as BMP-2 and BMP-7, TGF-B, platelet rich plasma and stem cells is being researched [14].

## Evolution of bone lengthening devices

Limb lengthening devices have evolved in the last 100 years. The first trials just used skeletal traction. The unilateral fixator was the standard method of fixation for a long time. The advances in fixator design included the application of half pins in more than one plane and addition of hinges which allowed joint movement during distraction [15–18]. Then, with the advent of Ilizarov’s revolutionary ideas, the principle of the circular fixator spread all over the world. The invention of the hexapodal frame had similar results and introduced the ability of lengthening and managing all deformities simultaneously without the need to change the frame. Computer assisted correction with the Taylor Spatial Frame, which is formed of two rings and six struts (each one connected with two universal hinges), was a real step forwards in improving the accuracy of lengthening and deformity correction [19]. In order to shorten the external fixation period, other methods were developed, such as lengthening over a small diameter nail and lengthening followed by nailing or plating. In children, flexible intramedullary nails were used to avoid physeal injuries. Over time, the incidence of fracture in the regenerated bone after removal of external fixation was reduced [20–23]. Finally, in the last two decades, internal bone lengthening nails without the need for external fixation have become popular. The Albizzia nail was designed by Guichet; it has a ratchet assembly and limb rotation is required to induce distraction [24]. In the United States, the ISKD (Intramedullary Skeletal Kinetic Distractor) was cleared for marketing in 2001. However, follow-up revealed a high rate of complication due to uncontrolled distraction and it was withdrawn from the market [15, 25, 26]. Currently, the motorized lengthening nails Fitbone and PRECISE, which do not require rotation for distraction, are becoming popular [27–31].

## Indications for limb lengthening

**Fig. 3:**
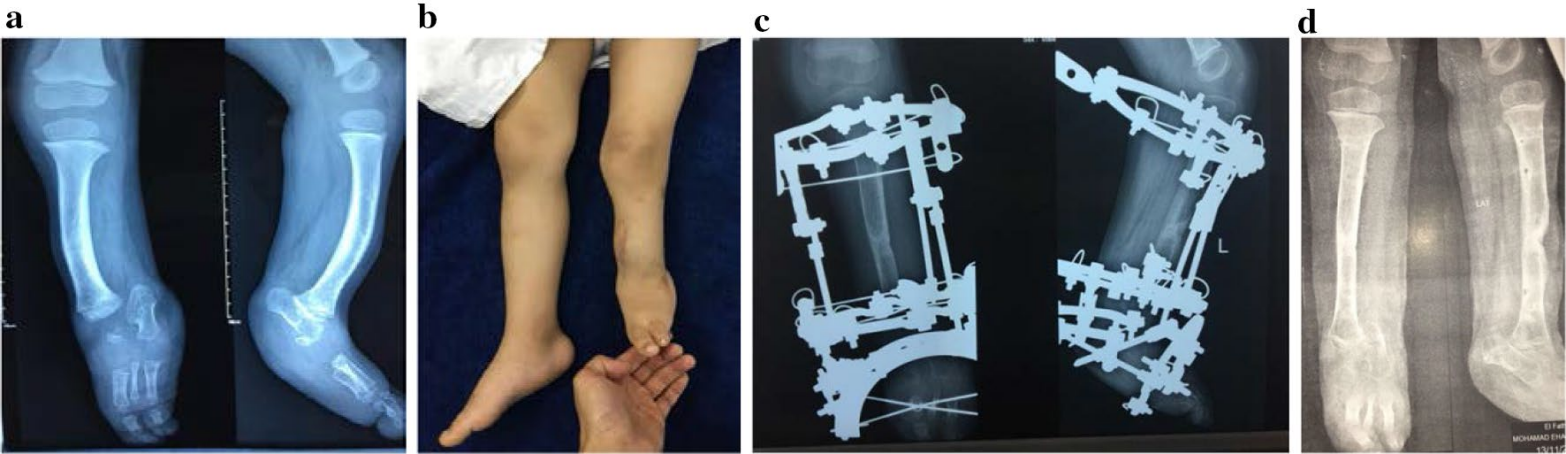
**a** A 3-year-old boy, fibular hemimelia, tibial shortening and angulation. **b** Clinical photo. **c** Picture at the end of lengthening and deformity correction. **d** Picture after fixator removal

In general, the indications for limb lengthening are controversial. Classic teaching classifies shortening into 3 categories: less than 2 cm, which can be ignored; 2–4 cm, with the possibility of lengthening; and more than 4 cm where lengthening is needed to avoid possible complications of lower limb length inequality such as pelvic obliquity and scoliosis. Also, a discrepancy of about 5 cm between leg lengths can be treated by epiphysiodesis in growing legs, or shortening of the longer leg at an appropriate time. However, this classification did not take into consideration the patient’s height, heel size, tolerability of the shoe lift, family opinion and psychological aspects. With growing experience of the new advances in limb lengthening, these factors usually play an important role in decision making [32, 33]. The aetiology of bone shortening and associated deformities is important for planning. The cause may be congenital deficiencies such as fibular hemimelia (Fig. 3), tibial hemimelia or congenital short femur, old poliomyelitis, bone tumours such as hereditary multiple exostosis [34] or past trauma. Bilateral lengthening may be indicated in cases of dwarfism caused by achondroplasia, especially if it is accompanied by deformities such as genu varum.

## Complications

Results of limb lengthening are significantly affected by the clinical experience of the operating surgeon [35]. Perhaps this is what causes the wide range of frequency of complications in the English literature. Even the classification of the type of complications varies widely, whether major or minor, simple or complicated, or affecting the final outcome [36, 37]. Many reports describe relatively small numbers of patients operated on by many surgeons with variable clinical experience over long time periods and sometimes in many centres [38–41]. It is difficult to reach valid conclusions when only a few patients are operated on every year (Table 1) [26, 36, 38–43]. The Ilizarov method causes postoperative, not operative, problems. The patient may stay in the frame more than 1 year. Most teaching courses and programmes only teach frame application, which is just the start in a long course of treatment.

**Table 1:** Overall complication rate during lengthening

Study	Procedure	No. of patients	Period of study	Complication rate	No. of surgeons
Bukva et al. [38]	IEF or IEF& IA	73	14 years	83.6%	Single
Lascombes et al. [36]	Multiple	32	3 years	75%	Multiple
Lee et al. [68]	ISKD	35	2 years	74%	Single
	PRECICE 1	34	Not available	17.4%	Single
	PRECICE 2	64	Not available	50%	Single
Castelein and Decquer [39]		39	14 years	84%	Multiple
Reitenbach et al. [40]	TSF & IEF	43	16 years	TSF 12.1% IEF 50%	Multiple
Manggala et al. [41]	TSF & IEF	14	4 years	TSF 28.6% IEF 71.4%	Multiple
Guerreschi and Tsibidakis [43]	IEF	63	25 years	81%	Multiple
Novikov et al. [42]	IEF	138	23 years	37%	Multiple

*IEF* Ilizarov external fixator, *IA* intramedullary alignment, *ISKD* Intramedullary Skeletal Kinetic Distractor, *TSF* Taylor Spatial Frame

The most common complication of external fixation is pin track infection, with a variable incidence which may reach 100% of treated patients. There are many variables which affect the frequency of this complication, such as duration of fixation, material of the wires or half pins, surgical procedure and wound care. Many pin site care programmes are designed to prevent the development of infection but are not supported by reliable evidence. Treatment usually starts with oral antibiotics and increasing the frequency of pin site cleaning in mild cases and ending up with removal of the pin in severe cases. The use of hydroxyapatite coated pins can reduce the incidence of pin site infection significantly [44].

Poor regeneration is a serious problem during limb lengthening and results from many systemic or local causes. It is important to modify the rate and frequency of distraction according to regeneration. Once delayed regeneration has been diagnosed, alternate cycles of compression distraction can solve the problem [45].

**Fig. 4:**
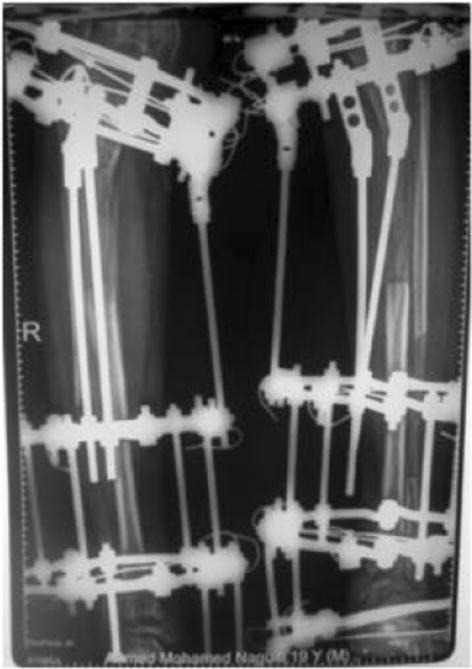
Malalignment of the tibia during distraction and application of hinges to correct it

Axial malalignment can develop with distraction as there is variable resistance of the different muscles surrounding the limb (Fig. 4). This can be corrected by changing the connecting rods of the construct in the outpatient clinic, using hinges. Joint subluxation or dislocation is a serious complication with increasing likelihood with unstable joints such as in congenital shortening. Management of joint abnormality or instability has to precede lower limb lengthening, and sometimes extending the frame to cross over the joint can guard against the development of this complication; however, this increases the possibility of stiffness. Premature consolidation of the regenerated bone has been reported due to irregular distraction of the osteotomy, especially in children. The treatment may be re-osteotomy or continuation of the distraction until the building force exceeds the resistance of the consolidation and the osteotomy opens once again, with severe pain. The resultant gap has to be closed and distraction starts again after a few days.

The incidence of complications is affected by the aetiology of the shortening and magnitude of the regenerated area. Extensive bone lengthening can adversely affect growth in children and increase the possibility of joint contractures [46, 47].

## Achondroplasia

Achondroplasia is the most common skeletal dysplasia characterized by disproportionate dwarfism. The strategy of lengthening may be transverse, including both tibias, or both femurs, in each stage [48, 49]. Most authorities adopt the transverse strategy as the patient can stop lengthening at any stage of treatment. The deformities seen in achondroplasia can be corrected simultaneously; lumbar hyperlordosis with an extension osteotomy of the femur, varus deformity of the leg, and a disproportionately long fibula may be reduced to normal length during the lengthening process.

**Fig. 5:**
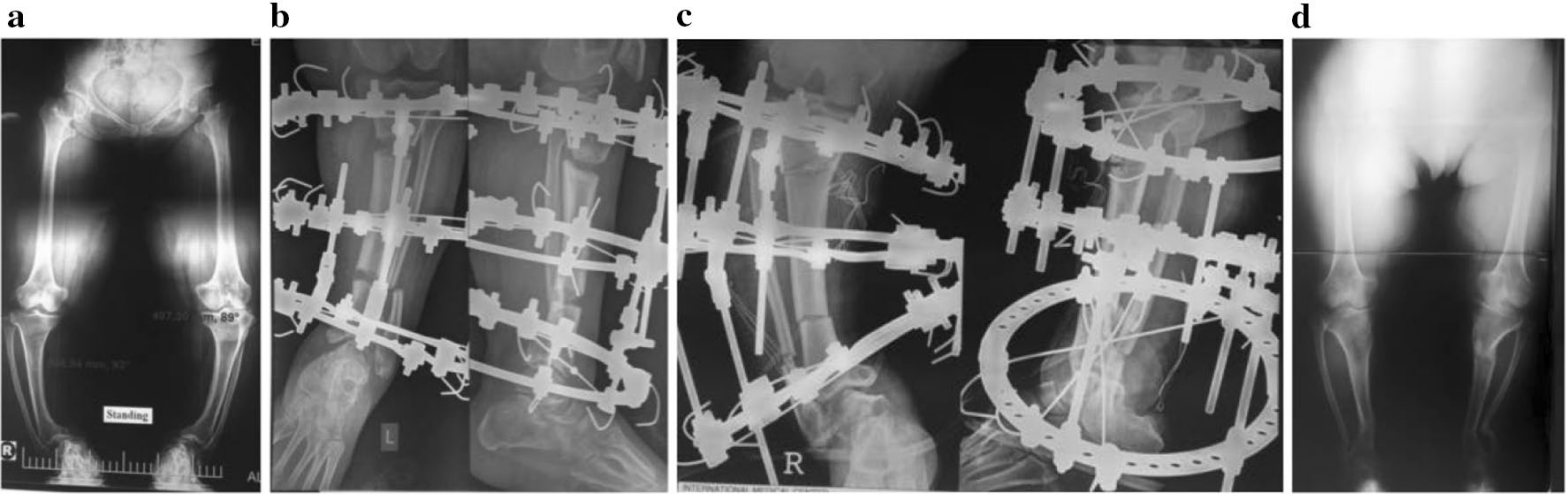
**a** 53-year-old female with achondroplasia with knee and ankle deformities. **b** Bifocal corticotomy and application of Ilizarov Frame on the left side. **c** Bifocal corticotomy and application of Ilizarov Frame on the right side. **d** After fixator removal

The soft tissues in achondroplasia are usually redundantly long [50]. The magnitude of lengthening usually ranges between 25 and 30 cm which can be gained after stages or extensive lengthening. In cases with knee and ankle deformities, bifocal tibial lengthening (Fig. 3) can restore the normal mechanical axis and achieve more lengthening with less time in the fixator [51–53]. In our institution, our protocol starts early, between 4 and 6 years, by differential tibial lengthening for a short distance to correct the varus deformity and get the fibular head in position with indirect tightening of the lateral ligament. Femoral lengthening is performed between 9 and 11 years. Then another tibial lengthening (mostly bifocal) starts at age 13–14. Finally, humeral lengthening is done at an age of 15 or 16. However, many patients are referred to our institution late and consequently we cannot follow this protocol (Fig. 5).

## Cosmetic lengthening

Bone lengthening for aesthetic reasons for normal or short stature has been reported recently. The ethical principles and psychological factors have to be taken into consideration. Psychiatric evaluation is mandatory for all patients to exclude body dysmorphic disorder [54]. It is mandatory to have detailed preoperative psychological analysis to rule out any psychiatric illness which might affect the patient’s ability to make a sensible decision. A single counselling session of limited time may not be enough to have fair appraisal of patient sanity. It would be wise to arrange several meetings between the patient and previously treated patients as part of the preoperative preparation programme to give them a real example of the difficulties to be expected before reaching their goal [55].

The first method used for cosmetic limb lengthening was the Ilizarov method, with a high rate of self satisfaction and improved level of social activities (96.7% of patients) [56]. Bilateral tibial lengthening, monofocal or bifocal, was the most common procedure, with a few cases having femoral lengthening as well. Trunk limb proportions may limit the magnitude of lengthening to 5–7 cm. In 2014, Novikov et al. published the largest series of cosmetic lower limb lengthening treated by Ilizarov apparatus at the Ilizarov institute, including 131 patients. The ages of patients ranged from 16 to 67 years, with a mean lengthening of 6.9 cm. At last follow-up there was one poor result (0.77%) with a rate of complications about 37% [42]. The authors were able to manage most of the complications successfully without affecting the final results. However, the patients were kept in the hospital for the whole period of treatment, allowing close monitoring and early management, which is not available in other institutions [55]. The time in the fixator was reduced by using the lengthening over nail technique with a rather moderate rate of complications [57, 58]. Intramedullary limb lengthening has developed as an alternative to external fixation which is quite attractive to patients; it has a lower rate of complications and higher costs [30]. Recently, there has been a considerable desire for cosmetic lengthening surgery around the world. In spite of the extensive experience of the treating surgeon, many soft tissue and bone problems are possibly expected. Safety of the patient has to be more important than gaining more length [42]. For example, if a weak regeneration zone develops, which is not responsive to cycles of compression distraction, the surgeon has to reduce the expected area of lengthening by gradual compression to improve the regeneration and avoid nonunion. In our opinion we think that this procedure has to be undertaken by a surgeon with great experience in the field to handle the potential complications.

## Upper extremity lengthening

**Fig. 6:**
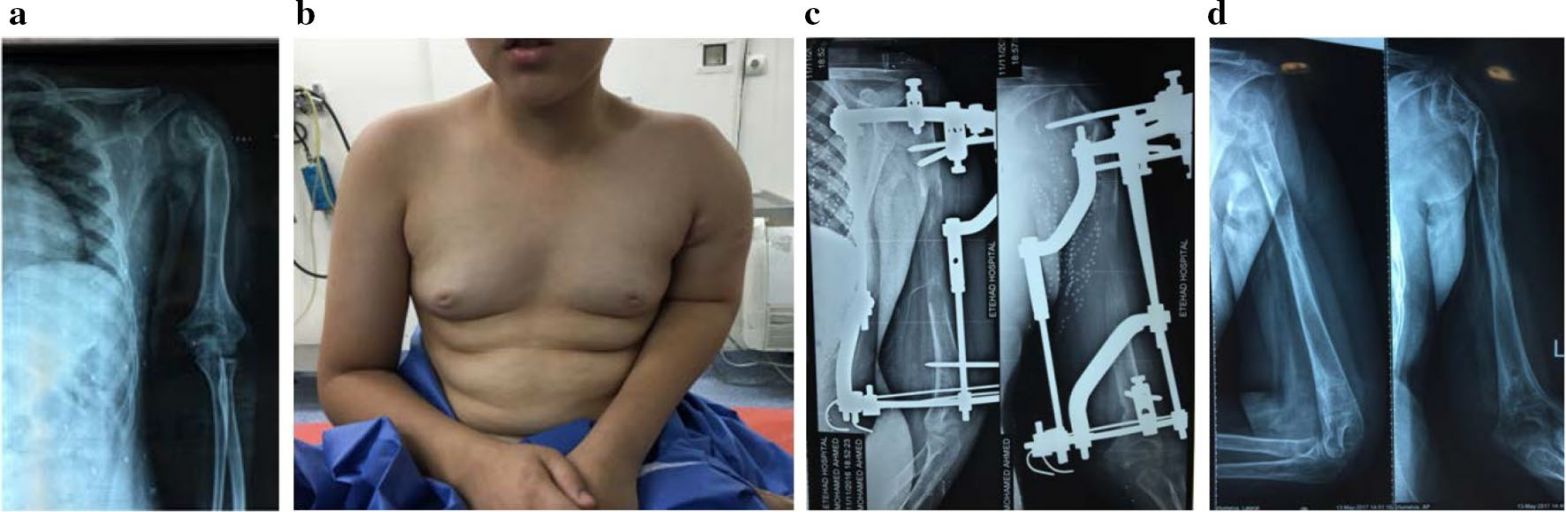
**a** A 12-year-old boy with 10-cm post-traumatic humeral shortening. **b** Clinical photo showing left humeral shortening. **c** Plain X-ray at the end of distraction. **d** Plain X-ray after fixator removal

There are few indications for upper extremity lengthening, but they include achondroplasia, hereditary multiple exostosis with shortening of the forearm bones, physeal growth arrest, amputation, infection and shortening from trauma. The reason upper extremity operations are not attempted as often as lower extremity operations might be due to reports of a high rate of complications and the possibility of functional deterioration [59, 60]. However, with developing experience we think that bone lengthening has no harmful effect on the upper extremity. Hybrid fixation minimizes the incidence of neurovascular injury (Fig. 6). Increasing the magnitude of lengthening in the lower limb more than 20% of the original bone length mostly raises the incidence of complications. However, we did not face this problem with lengthening up to 100% of the limb length in upper extremities. Preoperatively, there was some sort of abnormality of the shoulder joint as dysplasia of the articular surfaces in unilateral cases which did not affect the final outcome. Bracing for 1 month after fixator removal was advised to guard against fracture of the regenerated bone [61, 62]. Intramedullary lengthening nails were successfully applied for humeral lengthening in 6 cases [63]. The primary indication for forearm lengthening is discrepancy between the radius and ulna, congenital longitudinal deficiency and trauma. The rate of distraction has to be modified according to the degree of regeneration to avoid the reported complication of delayed bone formation [64, 65]. There are a few papers in the English literature reporting a small number of cases of short bone lengthening. Distraction histeogenesis (callotasis) was applied in a single stage and gradual lengthening for congenital and traumatic phalangeal shortening or amputation was achieved with excellent outcomes [66]. Two-stage treatment was also used including osteotomy and gradual distraction followed by bone graft [67].

## Conclusions

Limb lengthening is a rapidly developing field of orthopaedic surgery. Currently it is a standard procedure with predictable results, and indications have been extended to include the upper extremities and cosmetic lengthening. I think experience has a great impact on the results of the different procedures because follow-up and management of expected complications are cornerstones of treatment strategy. Unfortunately, the English literature has many papers with relatively small numbers of patients operated on by many surgeons over a long period. This means that the experience of the individual surgeon is based on only one or a few cases per year. Sometimes it is difficult to get valid conclusions from the reported mixed data. In spite of the introduction of the promising intramedullary lengthening nails and computer assisted external fixation, we still count on Ilizarov’s biologic laws. Advances through research to stimulate regeneration and reduce the period of treatment will be the real revolution in limb lengthening surgery.

## Data Availability

Not applicable.
